# Commentary: Raw Cow Milk Consumption and Atopic March

**DOI:** 10.3389/fped.2021.684662

**Published:** 2021-06-08

**Authors:** Laura Carucci, Serena Coppola, Rita Nocerino, Lorella Paparo, Carmen Di Scala, Roberto Berni Canani

**Affiliations:** ^1^Department of Translational Medical Science, University of Naples “Federico II”, Naples, Italy; ^2^ImmunoNutritionLab at the CEINGE Advanced Biotechnologies Research Center, University of Naples “Federico II”, Naples, Italy; ^3^European Laboratory for the Investigation of Food-Induced Diseases, University of Naples “Federico II”, Naples, Italy; ^4^Task Force on Microbiome Studies, University of Naples “Federico II”, Naples, Italy

**Keywords:** allergic march, food allergy, anaphylaxis, infant formula, immunonutrition

We have appreciated the interest of Dr Baars et al. in our paper describing dietary prevention of atopic march (AM) in children affected by cow milk allergy (CMA) (1). They claimed a lack of information on raw cow milk (unpasteurized cow milk) in our paper. In support of this point, they mentioned the result of a pilot study involving nine CMA children (2) that were able to tolerate up to 50 mL of raw milk (about 1,750 mg of cow milk proteins). This result was not confirmed by a similar study where five children with IgE-mediated CMA were orally challenged in a double-blind fashion with raw untreated cow milk, pasteurized cow milk, and homogenized/pasteurized cow milk. An extensively hydrolysed casein formula served as placebo. All patients presented significant allergic reactions from the consumption of the above three types of milk, whereas no adverse reactions to placebo were observed. The authors concluded that children with CMA cannot tolerate raw or pasteurized milk (3). Although, selected components of raw milk may potentially influence the immune system, proof based on controlled studies in children are still lacking (4). The authors of the PARSIFAL study concluded that raw cow milk may contain numerous disease-causing pathogens and that consumption of raw milk cannot be recommended as a preventive measure for allergy (5). Accordingly, none of the claims made by the raw milk advocates (including the postulated preventive effect against allergy) withstand the FDA scientific scrutiny (6).

CMA is one of the most prevalent food allergies and a major cause of anaphylaxis in childhood. Deaths from CMA-induced anaphylaxis have occurred in allergic children. Besides their effects on physical health and quality of life, CMA impose substantial economic burden on families and on healthcare system (7). The treatment following the diagnosis of CMA is complete avoidance of cow milk and foods containing cow milk proteins.

There is no solid evidence suggesting potential positive effects of raw milk in stimulating immune tolerance and in preventing the occurrence of AM in children with CMA. The use of raw milk in the dietary approach to CMA children is not recommended by current guidelines provided by scientific societies EAACI, DRACMA, NICE, ESPGHAN, NIAID, BSACI, and AAP (8, 9). Unfortunately, the false “health benefits” claims of raw milk may cause parents of children with CMA to give raw milk to their babies, that are most at risk for becoming ill or even dying from foodborne illness as a result of consuming contaminated raw milk (6). Fortunately, governmental agencies prohibit the consumption of raw cow milk especially for vulnerable groups (including infants and children) (9) for the risk of contamination with pathogens (*Listeria, Salmonella, Campylobacter, Enterohemorrhagic, and Shigatoxigenic Escherichia coli*) (10, 11).

Cow milk allergy presents many problems for patients, their families and national health care systems, we sincerely do not believe that it is worth adding other dangers in the absence of even minimal evidence of efficacy in modulating CMA disease course.

## Author Contributions

LC analyzed literature, wrote and read the manuscript. RBC designed and structured the paper, wrote and read the manuscript. SC, RN, LP, and CDS analyzed literature and read the manuscript. All authors listed have made a substantial, direct and intellectual contribution to the work, and approved it for publication.

## Conflict of Interest

The authors declare that the research was conducted in the absence of any commercial or financial relationships that could be construed as a potential conflict of interest.
